# Signaling the differences between cilia

**DOI:** 10.7554/eLife.12760

**Published:** 2015-12-23

**Authors:** Polina Lishko, Yuriy Kirichok

**Affiliations:** 1 Department of Molecular and Cell Biology, University of California, Berkeley, Berkeley, United Stateslishko@berkeley.edu; 2 Department of Physiology, University of California, San Francisco, San Francisco, United Statesyuriy.kirichok@ucsf.edu

**Keywords:** motile cilia, patch clamp, ion channels, calcium, Mouse

## Abstract

Calcium ion channels that determine many of the properties of cilia are different in motile cilia as compared to primary cilia and flagella.

**Related research article** Doerner JF, Delling M, Clapham DE. 2015. Ion channels and calcium signaling in motile cilia. *eLife* **4**:e11066. doi: 10.7554/eLife.11066

**Image** Motile cilia on the surface of an ependymal cell

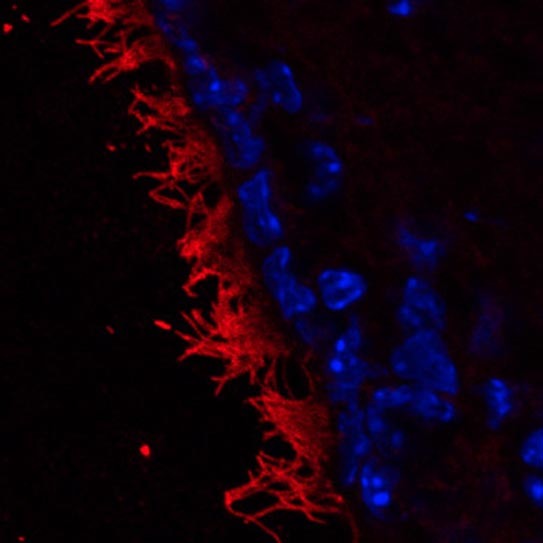

Almost all eukaryotic cells have hair-like projections called cilia on their surface. Cilia sense environmental cues, play crucial roles in organ development, and are responsible for moving cells and fluids. Problems that affect how cilia work cause a diverse set of ‘ciliopathies’, including polycystic kidney disease, infertility, airway diseases and hearing defects (Fliegauf et al., 2007). But how do cilia differ among various cell types?

Calcium ion (Ca^2+^) signaling is central for controlling how cilia move and respond to signals received from outside the cell (Phua et al., 2015). Now, in eLife, Julia Doerner, Markus Delling and David Clapham at Harvard Medical School and Boston Children’s Hospital demonstrate that the key principles of Ca^2+^ signaling differ significantly between different types of cilia (Doerner et al., 2015).

Cilia are categorized as either primary cilia or undulipodia based on obvious structural differences (Figure 1). Primary cilia are not able to move themselves, but help cells detect external cues and serve as a signaling hub during development (Goetz and Anderson, 2010). In contrast, undulipodia are able to move themselves and are subdivided into motile cilia that move fluid over the outer surface of the cell, and flagella that propel the cell through fluids (Fliegauf et al., 2007). Ciliary movement is directly associated with the axoneme – a structure made of microtubules that serves as a ‘skeleton’ and extends along the length of the cilium. All primary cilia contain nine pairs of microtubules that are arranged in a circle to give the so called 9+0 structure. Undulipodia have two additional microtubules in the center of the axoneme (the 9+2 arrangement; Figure 1).

**Figure 1.**
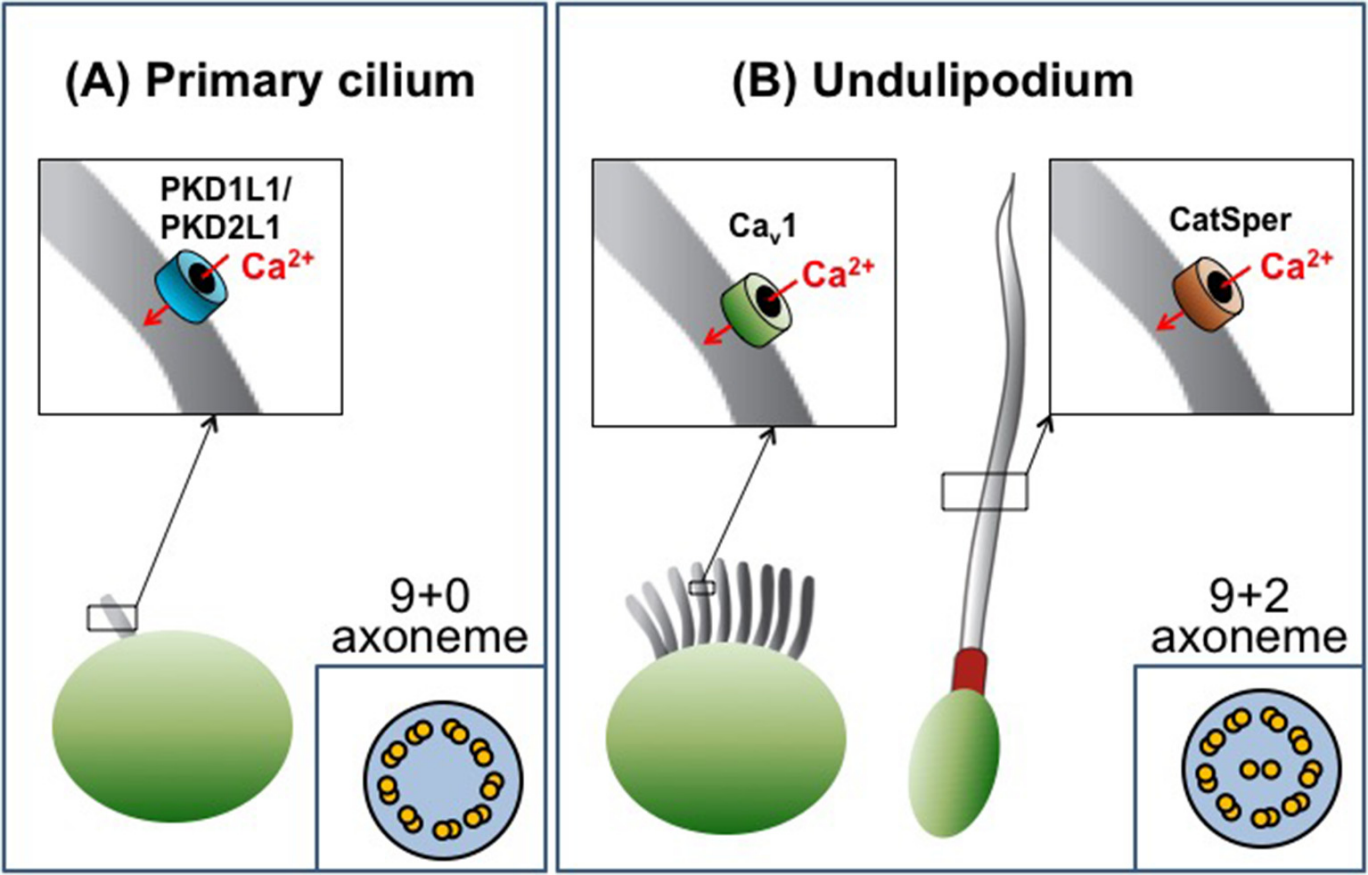
The diversity of cilia. There are two types of cilia, primary cilia and undulipodia, which are distinguished by differences in the structure of their axoneme. The level of calcium ions (Ca^2+^) inside the cilia is regulated by specialized ion channels. (**A**) The main Ca^2+^ channel for primary cilia has been identified as the PKD1L1/PKD2L1 complex. (**B**) There are two types of undulipodia: motile cilia and flagella. The main Ca^2+^ channel of mammalian flagella was identified as a protein called CatSper in 2013. Doerner et al. have now identified an L-type voltage-gated Ca^2+^ channel (Ca_v_1 family) as the main Ca^2+^ channel for motile cilia.

Beyond these obvious differences in structure, differences in the molecular pathways that control how the cilia work remained largely unknown. Direct investigation of the molecular composition of cilia is challenging, mainly due to their small size, the extremely low volume of ciliary cytosol, and the tight structural associations between various molecular components. Together, these adverse factors complicate the application of all biochemical, electrophysiological, or optical analytic methods.

However, since 2001 the Clapham lab has been instrumental in using the patch-clamp technique to study various ciliary structures, starting with the discovery of a mysterious channel-like protein located in the sperm flagellum (Ren et al., 2001). This protein, called CatSper, is structurally similar to the voltage-gated Ca^2+^ channels found in neurons. However, in the CatSper channel four individual subunits work together to form a functional channel, whereas in neuronal channels the four subunits are linked into a single molecule. Applying the whole-cell patch-clamp technique to sperm cells revealed that CatSper is only weakly voltage-dependent, defying expectations. Instead, its activity is controlled by the pH inside the cell (Kirichok et al., 2006) and by the female steroid hormone progesterone (Lishko et al., 2011; Strunker et al., 2011). CatSper is now established as the principal, and perhaps only, Ca^2+^ channel in the flagella of sperm cells and is, therefore, indispensable for normal sperm motility and male fertility (Figure 1).

In another technological and conceptual breakthrough in 2013, the Clapham group applied the patch-clamp technique directly to primary cilia and found that the main Ca^2+^ channel of this organelle consists of two subunits: PKD1L1 and PKD2L1 (DeCaen et al., 2013; Delling et al., 2013; Figure 1). These proteins belong to a group of proteins called polycystins, some of which are mutated in patients with polycystic kidney disease. In mice, PKD1L1 mutations disrupt left-right patterning and cause the developing embryos to die (Field et al, 2011). In contrast, PKD2L1-deficient mice can survive, but demonstrate intestinal malrotation – a condition that causes parts of the gut to develop in the wrong positions (Delling et al., 2013). The Clapham group demonstrated that PKD1L1-PKD2L1 channels in the ciliary membrane cause a resting Ca^2+^ concentration that is approximately five times greater in the primary cilia than in the cell body. Thus, primary cilia are a specialized Ca^2+^ signaling organelle, in which Ca^2+^ may be required for regulating sonic hedgehog and other signaling pathways.

Doerner, Delling and Clapham have now made the first patch-clamp recordings on motile cilia. The recordings were made on ependymal cells, which line fluid-filled spaces in the brain and spinal cord, and revealed several differences between motile cilia and other cilia types. Firstly, the abundance of Ca^2+^ channels in the plasma membrane of motile cilia appears to be much lower than in primary cilia and flagella. Only one type of Ca^2+^ channel was apparently present in the motile cilia of ependymal cells: a voltage-gated Ca^2+^ channel (in the Ca_v_1 subfamily). Moreover, the ciliary membrane contained many fewer of these channels than the plasma membrane of the rest of the cell (Figure 1).

Furthermore, Doerner et al. found that the resting Ca^2+^ concentration in the motile cilia was much lower than that in primary cilia. Finally, although the voltage-gated Ca^2+^ channels cause a substantial elevation of the Ca^2+^ concentration inside the cilium upon membrane depolarization, this does not measurably change ciliary motility. This is in striking contrast to the sperm flagellum, where motility is strongly regulated by the movement of Ca^2+^ ions through CatSper. However, a possibility still remains that in addition to voltage-gated Ca^2+^ channels, motile cilia contain other, yet unidentified, types of Ca^2+^ channels that would be more efficient in controlling ciliary beat.

The remarkable technical and conceptual advances made by the Clapham group should lead to the discovery of other ion channels and new regulatory pathways in these fascinating structures.
